# Placebo Effect on Modulating Empathic Pain: Reduced Activation in Posterior Insula

**DOI:** 10.3389/fnbeh.2020.00008

**Published:** 2020-01-31

**Authors:** Yili Zhao, Ruixuan Liu, Jianxin Zhang, Jing Luo, Wencai Zhang

**Affiliations:** ^1^CAS Key Laboratory of Mental Health, Institute of Psychology, Chinese Academy of Sciences, Beijing, China; ^2^Department of Psychology, University of Chinese Academy of Sciences, Beijing, China; ^3^Beijing Key Laboratory of Learning and Cognition, Department of Psychology, Capital Normal University, Beijing, China

**Keywords:** empathy for pain, picture-based paradigm, posterior insula, sensory area, placebo effect

## Abstract

Little evidence exists to confirm whether the sensory-related neural activity that occurs when observing others in pain is highly responsive to empathy for pain. From a perspective of intervention, the present study employed placebo manipulation with a transferable paradigm to explore whether the sensory regional activation that occurs when viewing pictures of others in pain could be modulated by the placebo effect. We first performed a screening behavioral experiment for selecting placebo responders and then entered them into a functional magnetic resonance (fMRI) experiment in which they were exposed to the same conditions as before. Participants were informed that it was equally possible to be assigned to the treatment group (placebo manipulation) or the no-treatment group (control); they all, in fact, received treatment and placebo effect would be detected by comparing placebo conditions and no-placebo control condition. Each participant experienced a phase of reinforcing placebo belief with pain in self and a phase of testing transferable placebo effect on empathy for pain. As a result, we found significant activation in sensory areas, including the posterior insula (PI) and the postcentral gyrus, and in the middle cingulate cortex while participants observed pictures of others in pain. More importantly, for the first time, we observed relieved activation in the PI modulated by the placebo effect only associated with pain pictures but not with no-pain pictures. This suggests that sensory activity in the PI might be involved in the processing for empathic pain. This new approach sheds light on research and applications in clinical settings.

## Introduction

Individual experience tells us that, when seeing others in pain, we seem to not only understand their situations but also share their feelings of pain to some extent. The pain developed from observing actual or threatened tissue damage in another person is called empathy for pain (Zaki et al., 2016). It is believed that empathy for pain has a certain relationship with the direct experience of pain: evidence has shown that direct pain and empathic pain interact with each other (Vachon-Presseau et al., 2011; Reicherts et al., 2013; Hurter et al., 2014), and some overlapping brain regions have been confirmed (Singer et al., 2004; Gu et al., 2010; Osborn and Derbyshire, 2010; Valentini, 2010). These findings suggest that shared psychological representations may be involved in both direct pain and empathic pain. However, empathic pain may be a double-edged sword. In the case of perceiving others’ suffering, the psychoneural resonance in pain-processing areas between other and self may trigger empathic concern, but the same signals may also constitute a threat to the individual that can lead to personal distress. This distress can be costly, both physiologically and cognitively, and can eventually conflict with the observer’s capacity to be of assistance to the other, therefore should be alleviated (Decety, 2011).

In line with previous studies, the experience of direct pain: (a) can be coded for pain localization, quality, and intensity, processed by both the primary and secondary somatosensory cortices (S1, S2) and by the posterior insula (PI); and (b) can be coded for both the unpleasant or distressing experience of pain and the drive to terminate such an experience, processed by the midcingulate cortex (MCC), anterior insula (AI) and the amygdala (Price et al., 1987; Treede et al., 1999; Neugebauer et al., 2004; Bernhardt and Singer, 2012; Eisenberger, 2015). Considerable studies have reported the MCC and AI, which are considered to represent similar affective characteristics to direct pain, to be activated during empathy for pain (Singer et al., 2004; Gu et al., 2010; Valentini, 2010). Although areas involved in the sensory discriminative dimension of pain (S1, S2 and the PI; Decety, 2011) were detected as well when observing others in pain (Avenanti et al., 2005; Bufalari et al., 2007; Moriguchi et al., 2007; Danziger et al., 2009; Osborn and Derbyshire, 2010), some researchers considered that they might just be a non-specific activation, based on both the perception of touch and of body parts movement (Keysers et al., 2010; Bernhardt and Singer, 2012). Thus, further experimental evidence is needed to clarify whether the activities in sensory areas are highly responsive to the perception of pain in others. which should be obtained in a stricter control experimental setting. Most previous studies have only observed the changes in brain activation in the above-mentioned areas of empathy for pain by comparing painful and non-painful conditions. Few studies observed them from the perspective of the modulation of empathic pain. With the paradigm of modulation, brain activation of empathy for pain could be observed both by comparing the control and modulation conditions for the painful condition and by comparing painful and non-painful conditions. These brain changes could be more definitively explained by the differences of empathic pain experiences in multiple conditions.

In the present study, we aimed to use placebo manipulation to modulate empathic pain, considering its advantages in several aspects. First, the placebo effect works by manipulating expectations towards a specific target. Wager et al. (2004) found that analgesic placebo manipulation could lower the activation in sensory areas only when intense shocks were delivered, whereas no changes occurred with mild shocks. Similarly, other investigators revealed that anxiolytic placebo manipulation reduced activation in regions related to emotion processing when participants observed unpleasant pictures instead of neutral pictures (Zhang et al., 2011), suggesting that the placebo effect might only work when the target was relevant for survival and human well­being (Wager and Atlas, 2015). Furthermore, placebo manipulation was designed to show altered brain activation between the original level and modulated level (e.g., high vs. low) of empathy for pain without changing the content of existing stimuli. In this case, there were neural components highly responsive to empathic pain and fewer unexpected neural components compared with observed altered brain activation between two different stimuli contents (e.g., pain vs. no pain). In contrast, if reduced activation in one region is observed when comparing pain with neutral pictures, one cannot judge whether the activation is closely related to pain content, or to other irrelevant components, as the contents in the two situations varied. Finally, compared with other techniques, for example, cognitive reappraisal, placebo manipulation seems to recruit fewer cognitive components applied for modulation. A previous study indicated that the placebo effect was as equally effective in reducing negative arousal as cognitive reappraisal when participants were instructed to use the two strategies to observe unpleasant pictures, without necessarily mobilizing large amounts of dorsal lateral prefrontal cortex (DLPFC) activation, which is specifically engaged in the process of cognitive appraisal (Zhang et al., 2013). These findings support the idea that placebo manipulation is a suitable tool to be applied in our study for modulating brain activation related to empathic pain.

In terms of experimental paradigms to research empathic pain, Lamm et al. (2011) have classified previous experiments on empathy for pain into picture-based paradigms (showing pictures of body parts receiving pain) and cue-based paradigms (employing abstract visual symbols to signal pain in others). The meta-analysis from the same study demonstrated that generally, the picture-based design induced much greater activation in somatosensory regions when compared to the cue-based design. Based on the cue-based paradigm, Rütgen et al. (2015) utilized placebo analgesia manipulation and tested whether seeing others in pain was also influenced by relieving pain in self. They observed decreased activation in the anterior midcingulate cortex (aMCC) as well as the AI in both the pain in self and in other conditions in the placebo group. However, they did not show whether somatosensory regional activation also reduced others’ pain by the placebo analgesia effect. We argue that the failure to observe decreased somatosensory activity could be largely attributed to the application of the cue-based paradigm. For example, one research studied how cumulative experiences of social discrimination impact brain response during empathic responding, they found lack of significant dACC and aINS activation and considered this case might be explained by the nature of the task which is more dependent on cognitive perspective taking or on affective sharing (Fourie et al., 2019).

To overcome these problems, first, in order to elicit reliable activation of sensory areas we applied the picture-based paradigm in this study. To get a robust placebo effect on empathy for pain, we referred to the indirect placebo effect paradigm proposed by Zhang et al. (2011), in which the placebo belief was configured in direct pain first and then transferred its effect to negative emotion. The results demonstrated significantly increased activation with placebo treatment in the subgenual anterior cingulate cortex (sgACC)/orbital frontal cortex (OFC), a region which has been shown to engage in placebo anticipation (Wager et al., 2004), and decreased activation in the amygdala, insula, and dACC when viewing unpleasant pictures (Zhang et al., 2013). Based on this idea, we developed our experiment by first forming the placebo belief in direct pain and then transferring this effect to empathy for pain. Several additional strategies were also employed in the placebo effect design, including a cover story of a “random-controlled” assignment to improve the reliability of the experiment, a within-group design to minimize the influence of individual differences, a two-round training to reinforce placebo belief, and a former behavioral experiment to screen out placebo responders for the functional magnetic resonance (fMRI) experiment. Three questions are addressed in this article: (1) whether significant activation in sensory areas (e.g., S1, S2 and the PI) could be observed in the network of empathy for pain? (2) If the answer is yes, whether the induced sensory regional activation when seeing others in pain could be alleviated by the placebo effect? And (3) whether sgACC and adjacent OFC activations would take part in placebo modulation?

## Materials and Methods

### Participants

The sample size was calculated by simulation based on R (R Development Core Team, 2011). RStudio^1^ was used to run the custom R script to perform the analysis. In order to estimate the effect size, we referred to a previous study in our lab that had a similar design (Zhang et al., 2011). Since the value of Cohen’s *d* in that study is quite large (bigger than 1.5), we set Cohen’s *d* value as 0.8, which is still large but more reasonable. The within-subject correlation was set as 0.7, according to results of the same study. Based on the estimated parameters above, we generated 3,000 bootstrap samples and the result showed that to reach 80% power we needed at least a sample size of 17.

First, we conducted a behavioral experiment to select the placebo responders as the final individuals participating in the fMRI experiment. Evidence from previous research indicates that the placebo effect is relatively reproducible when the contexts remain consistent (Whalley et al., 2008; Morton et al., 2009). Forty-eight participants (female = 33, mean age = 23.10 years, standard deviation = 2.34) were enrolled and informed that there was a chance they would enter a subsequent fMRI experiment 1 month later. Ultimately, 24 participants were identified for the fMRI experiment (female = 18, mean age = 22.88 years, *SD* = 1.96). This study was approved by the ethics committee of the Chinese Academy of Science; prior written informed consent was obtained from all participants. None of the participants had a history of neurological, psychiatric, or major medical disorders.

### Materials

All pain stimuli used in both the behavioral and fMRI experiments were delivered by a CO_2_ laser stimulator (Precise Laser-DM 300, China) with a 2.5 mm spot diameter and a 100 ms pulse duration. Stimulation was applied to the dorsum of the right hand within a 3 cm × 3 cm square, with each stimulus applied to a different spot to avoid habituation. The distance from the laser probe to the skin surface was 9 cm. Participants were exposed to individually calibrated high or low painful stimulation, in which the output energy was kept between 200 and 350 mJ to prevent skin damage. The average intensity for high and low painful stimuli was respectively 317.27 ± 23.05 mJ and 223.71 ± 17.84 mJ.

**Figure 1:**
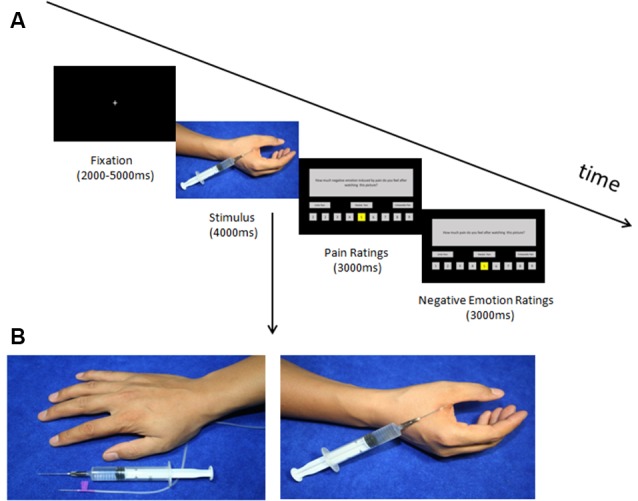
**(A)** Timeline of the trials in the test phase and **(B)** two sample pictures. **(A)** Trial structure of the test phase. A fixation point appeared followed by the picture; after the picture disappeared the participants rated the pain intensity and negative emotion induced by watching the picture (within 3 s each). **(B)** In the no-pain condition, two needles (injection and intravenous) were placed next to the hands, whereas, in the pain condition, a needle was inserted into the hands.

Before the behavioral experiment, we conducted a picture validation. Twenty-five participants were recruited to rate 72 digital colored photographs taken by our lab members, in which someone’s body parts were shown either in painful or non-painful situations. In the pain pictures, one or two needles were injected into a person’s hand or foot (e.g., in the dorsum or palm of the hand); whereas in the control pictures, the needle was placed next to the hand or foot, without penetrating the skin (see Figure 1). All the participants were instructed to rate the degree of pain elicited in each picture on a scale from 0 to 100 (0 = non-painful, 100 = unbearably painful).

Thirty-six pictures were selected for the behavioral experiment, consisting of 18 pain pictures which got top pain scores (mean = 59.26, *SD* = 5.04) aimed to induce participants’ empathy for pain and 18 no-pain pictures which got bottom pain scores (mean = 12.64, *SD* = 1.91) were used as a baseline. In addition, 96 pictures which were adapted from the 36 pictures in the behavioral experiment by reversal and repetition were applied in the fMRI study, including 36 original pictures and 36 reversed ones by them, six repeated pictures with topmost scores of pain pictures and six reversed ones by them, and six repeated pictures with bottommost scores of no-pain pictures and six reversed ones by them. The newly formed pictures contained 48 pain pictures (mean = 59.60, *SD* = 4.75) and 48 no-pain pictures (mean = 12.39, *SD* = 1.81).

### Procedures

The procedure of the behavioral experiment, which focused on selecting placebo responders, was quite similar to the fMRI experiment. Prior to the behavioral experiment, all 48 participants were informed that they would participate in a double-blind clinical study, aiming at examining whether a new type of magnetic equipment could alleviate pain directed to the self and others. The working principle of the equipment was explained to be in accordance with the acupuncture theory of traditional Chinese medicine, that is, when the magnetic equipment worked on distinct acupoints, corresponding treatment effects would exert and act on the targeted body parts or mental problems. The working state (on and off) of the equipment was described to be controlled by an internal program compiled in advance. As a result, it was hard for participants to infer the working state of the equipment externally. The so-called treatment equipment was, in fact, a sham, and no effect was delivered at all. The experiment consisted of three main phases: a “calibration procedure” with pain stimuli, a “conditioning phase” to build up the placebo belief, and a “test phase” to measure the placebo effect on alleviating empathy for pain.

In the calibration phase, we tested the pain threshold individually to determine the personalized stimulus intensity. Participants were asked to rate those stimuli in an ascending as well as in a descending order, according to a 9-point scale ranging from 1 = “perceptible, but non-painful sensation” to 9 = “unbearable pain” and 5 = “moderate pain.” Finally, three stimuli consistently rated as 6–8 (painful, but bearable) and three stimuli consistently rated as 1–3 (perceptible, but non-painful sensation) were selected respectively as pain and no-pain stimuli.

The conditioning manipulation phase was conducted with “random-controlled” instructions. All participants were informed that they would be randomly allocated to either the treatment group or the control group. However, in truth, all participants were assigned to the treatment group and experienced treatment (placebo manipulation). Hence, all the participants completed the placebo and no-placebo control conditions in a within-group design and the placebo effect would be detected by comparing placebo condition and no-placebo control condition. That is, in this study, each participant would experience placebo condition and no-placebo control condition in sequence. Placebo condition was the “treatment” situation in which magnetic equipment (i.e., the placebo adopted in this study) was “on” and no-placebo control condition was the “no treatment” situation in which magnetic equipment was “off.” Here, we adopted an innovative approach including two steps to help each participant build up a reliable placebo belief in the treatment. First, all participants were informed they would be given a “real” treatment experience of direct pain. An electrode of the magnetic equipment was linked to their Hegu acupoints (i.e., on the dorsum of their hands) which acupoint represents an analgesic effect, according to traditional Chinese medicine. Participants were then informed they would receive two blocks of stimuli (five stimuli for each block) that they rated as painful in the calibration phase. Since the treatment equipment worked only during one of the blocks, participants felt pain alleviation under the treatment condition, whereas they experienced increased pain under the no-treatment condition. By comparing the different experiences on receiving treatment or not, participants would learn by themselves how it felt when there was an analgesic treatment. The actual manipulation of this step was that in one block while the equipment was “on” we delivered stimuli individually rated as no pain, whereas in the other block while the equipment was “off” we delivered stimuli rated as pain, for each subject. This step ensured that participants could learn the kind of analgesic experience during placebo treatment. The purpose of the second step was to help participants believe that they were allocated to the treatment group, by training. Participants were informed that they would receive another 10 blocks of pain stimuli (five stimuli of each) and were asked to judge which group (treatment or no treatment) they belonged to. Participants were instructed to make judgments by their own unique experiences on receiving treatment or no. The actual manipulation in this step was that for each participant we delivered five blocks of no-pain stimuli (placebo manipulation, abbreviated P) and five blocks of pain stimuli (no placebo control, abbreviated C), the 10 blocks were presented either in the order P-C-P-C-P-C-P-C-P-C or C-P-C-P-C-P-C-P-C-P. One order was applied to half participants and the other order was applied to the other half participants in a counterbalanced way. Following the learning of the analgesic experience induced by treatment in the first step, and the adequate repetitive learning of 10 blocks in the second step, as we expected, all participants judged they were in the treatment group and had experienced a real analgesic treatment.

Compared to most placebo effect studies, in which participants were directly notified that they would receive the treatment, the special “random-controlled” verbal instructions employed in our study have at least two advantages: to begin with, as the participants primarily believed to be taking part in a randomized-controlled study, their placebo belief was built based on the repetitive learning about their experience of “treatment” rather than the direct notification from the investigator. In this way, they would naturally believe that they were receiving analgesic treatment and were less likely to suspect the validity of the treatment. In addition, since participants were not directly informed whether they would receive the treatment or not, our design, to some extent, could avoid some ethical issues induced by open verbal deception existing in placebo clinical research.

In the test phase, we aimed at examining whether the placebo belief built in the direct pain condition could transfer to situations when seeing others in pain. Beforehand, we moved the electrode from the Hegu acupoint to the Quchi acupoint. Participants were told that the treatment on this acupoint could relieve their feelings of pain and reduce negative emotions arising when seeing others in pain. Thirty-six pictures of pain and non-pain were divided into six blocks, each block consisted of three pain and three no-pain pictures. The average ratings of pain intensity between each block were already controlled to be statistically equal. The order of the experimental conditions for each individual was the same as in the second step of the conditioning phase. After each picture was presented, participants were required to rate for pain intensity (1–9, 1–no pain, 9–unbearable pain) and negative emotion (1–9, 1–no negative emotion, 9–unbearable negative emotion) induced by seeing others in that situation. At the end of the behavioral experiment, we interviewed each of the participants and asked whether they considered the treatment effective. Finally, 24 placebo responders were selected in terms of their performance in the behavioral experiment and were recalled for the fMRI Experiment 1 month later. The procedure of the fMRI experiment was relatively identical to the behavioral experiment, except for the fact that the test phase of empathy for pain was conducted in the scanner. In addition, in the test phase, the number of pictures was increased to 96 and split into eight blocks, each containing six pain and six no-pain pictures. The pictures are visually presented to the participants by using an MRI-compatible projection system. One trial of the fMRI experiment is shown in Figure 1.

### Image Acquisition

Data were collected on a GE 3.0T Trio MRI scanner at the Chinese Academy of Sciences, Institute of Psychology. High-resolution T1-weighted structural images were acquired with a 3D gradient–echo pulse sequence (TR = 6.9 ms, TE = 3 ms, FA = 8°, FOV = 256 mm × 256 mm, matrix size = 256 × 256, slice thickness = 1 mm, Voxel size = 1.0 mm × 1.0 mm × 1.0 mm), Functional images were acquired using a T2*-weighted echoplanar imaging (EPI) sequence with 37 transverse slices covering the whole brain (TR = 2,000 ms, TE = 30 ms, FA = 90°, FOV = 220 mm × 220 mm, matrix size = 64 × 64, slice thickness = 3.5 mm, interslice gap = 0.5 mm, Voxel size = 3.0 mm × 3.0 mm × 4.0 mm).

### fMRI Data Processing and Analyses

All pre-processing and statistical analysis of the images was performed using Statistical Parametric Mapping (SPM8^2^). In the pre-processing, the first five functional EPI volumes were discarded to allow for the T1 equilibration. Subsequently, the remaining data were slice time corrected. Head motion correction was applied and individual structural images (T1-weighted MPRAGE) were co-registered to the mean functional image using a rigid-body transformation. Functional images were normalized into a standard anatomical space (3 × 3 × 3 mm isotropic vexes) based on the Montreal Neurological Institute (MNI) template. The resulting fMRI data were then spatially smoothed with an 8 mm FWHM Gaussian isotropic kernel. The imaging data of one participant, who met the exclusion criteria of 2.0 mm and 2.0° in maximum head motion, was deleted.

In the first-level analysis, to assess the neural activity corresponding to the processing of pain and no pain pictures under placebo and no-placebo control conditions, four separate regressors (PP, watching pain pictures in the placebo condition; PN, watching no pain pictures in the placebo condition; CP, watching pain pictures in the no-placebo control condition; CN, watching no pain pictures in the no-placebo control condition) were created for model specification. These regressors were time-locked to the onset of picture presentation and then convolved with a canonical hemodynamic function. Residual effects of head motion were corrected by including the six estimated motion parameters of each participant, which worked as regressors of no interest in the design matrix. A high-pass filter with a cut off frequency of 1/128 Hz was used to adjust for low-frequency components, and serial correlations were accounted for with an autoregressive AR (1) model.

In the second-level analysis, the relevant parameter contrasts generated on an individual level were submitted to a group analysis by using a random effect model. A 2 × 2 full factorial analysis of variance (ANOVA) was conducted with data from 23 participants (1 participant was excluded as a result of reaching the criteria of 2.0 mm and 2.0° max head motion, the behavioral data were deleted as well). A repeated-measures ANOVA, with two within-participants factors: CONDITION (placebo vs. no-placebo control) and PICTURE (painful vs. non-painful,) were applied to assess main effects and interactions. For specific regions of interest (ROIs) of insula, postcentral and orbital gyri, we applied a small-volume correction (SVC) on these ROIs with an anatomical mask according to the AAL template (Tzourio-Mazoyer et al., 2002) provided by WFU PickAtlas software (Version 3.0^3^). For the whole-brain analyses, results with a threshold set at *p* < 0.001 (voxel level, uncorrected), and two or more contiguous voxels were reported. For the SVC analyses, the threshold was set at *p* < 0.001 (voxel level, uncorrected, two or more contiguous voxels). All fMRI results were not corrected by FDR and FWE.

### Behavioral Measures Analyses

Participants’ ratings on both pain intensity and negative emotion of empathy for pain induced by watching the pictures were analyzed using SPSS 18.0 (IBM software). In the behavioral experiment, paired sample *t*-tests were performed to selected placebo responders. To explore whether the placebo effect on the selected placebo responders was reproducible and consistent, we used two linear regression analyses to detect whether the ratings of placebo responders from the prior behavioral experiment could predict those of the latter fMRI experiment. In the fMRI experiment, two repeated measure ANOVAs were conducted to detect if the placebo effect worked when watching the pictures with two within-subject factors, namely CONDITION (placebo vs. no-placebo control) and PICTURE (pain vs. no pain). Subsequently, a simple effect test was performed to explain the interaction effects.

## Results

### Behavioral Results

In the behavioral experiment for selecting placebo responders, 24 participants showed a significant placebo effect, in which they reported much higher ratings both on feelings of pain intensity (PI) and on negative emotion induced by seeing others in pain (NE) in the no-placebo control condition than in the placebo condition, *t*_(23)_ = 5.072, *p* < 0.001, Cohen’s *d* = 1.035, 95% confidence interval (CI) for Cohen’s *d* = (0.529, 1.527), and *t*_(23)_ = 6.664, *p* < 0.001, Cohen’s *d* = 1.360, 95% CI for Cohen’s *d* = (0.793, 1.912), respectively. Of these, no one reported the treatment as ineffective. Regression analyses found that, for the selected placebo responders, ratings of PI under the placebo condition (pain vs. no pain) from the behavioral experiment could significantly predict the PI ratings in the fMRI experiment (*F*_(1,22)_ = 19.210, *β* = 0.691, *p* < 0.001, adjusted *R*^2^ = 0.453), as well as the rating of NE under the placebo condition (pain vs. no pain) in the behavioral experiment. The predictability was also significant (*F*_(1,22)_ = 12.951, *β* = 0.618, *p* = 0.002, adjusted *R*^2^ = 0.352).

**Figure 2:**
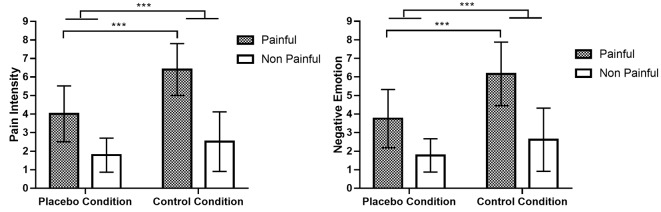
Ratings of pain intensity and negative emotion induced by pain and no pain pictures. Comparing placebo and no-placebo control conditions, pain pictures induced higher ratings of pain intensity and negative emotion in the no-placebo control condition compared with the placebo condition (*p* < 0.001), and the difference between pain and no pain pictures was also much higher in the no-placebo control condition than in the placebo condition (*p* < 0.001). ***p < 0.001.

In the test phase of the fMRI experiment, there was a significant main effect of CONDITION on ratings of PI, *F*_(1,22)_ = 43.606, *p* < 0.001, *η*^2^ = 0.665, 90% CI for *η*^2^ = (0.428–0.766), as well as on ratings of NE, *F*_(1,22)_ = 41.191, *p* < 0.001, *η*^2^ = 0.652, 90% CI for *η*^2^ = (0.410–0.757). Similarly, the main effect of PICTURE was significant on ratings of PI, *F*_(1,22)_ = 125.508, *p* < 0.001, *η*^2^ = 0.851, 90% CI for *η*^2^ = (0.723–0.896), and on ratings of NE (*F*_(1,22)_ = 95.596, *p* < 0.001, *η*^2^ = 0.813, 90% CI for *η*^2^ = (0.658–0.869). The interaction effect between CONDITION and PICTURE was significant both for ratings of PI, *F*_(1,22)_ = 29.856, *p* < 0.001, *η*^2^ = 0.576, 90% CI for *η*^2^ = (0.311–0.703), and for ratings of NE, *F*_(1,22)_ = 29.944, *p* < 0.001, *η*^2^ = 0.576, 90% CI for *η*^2^ = (0.312–0.704). Simple effect analyses revealed that for both PI and NE ratings, the difference between pain and no pain pictures in the no-placebo control condition was significantly smaller than that in the placebo condition, *t*_(22)_ = 5.457, *p* < 0.001, Cohen’s *d* = 1.138, 95% CI for Cohen’s *d* = (0.602, 1.658), and *t*_(22)_ = 5.478, *p* < 0.001, Cohen’s *d* = 1.141, 95% CI for Cohen’s *d* = (0.606, 1.664), respectively. The results are illustrated in Figure 2.

### fMRI Results

#### Brain Network When Seeing Others in Pain

**Figure 3:**
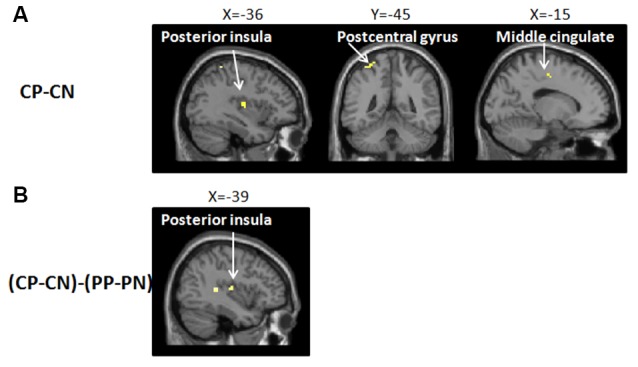
Brain network of **(A)** the main effect in the empathy for pain with CP-CN (i.e., watching pain pictures vs. watching no pain pictures, in the no-placebo control condition). In the no-placebo control condition, some brain regions, including the left postcentral gyrus (−36, −45, 63), left posterior insula (PI; −36, −12, 9), and midcingulate cortex (MCC; −15, −12, 48), showed greater activation in the pain pictures compared to the no pain pictures, *p* (unc.) < 0.001, two continuous or more voxels; **(B)** the interaction with (CP-PP)-(CN-PN) [i.e., (no-placebo control condition vs. placebo condition, when watching pain pictures) − (no-placebo control condition vs. placebo condition, when watching no pain pictures)]. The activation of the PI (−39, −15, 9) produced a greater attenuation for pain pictures vs. no pain pictures when comparing the no-placebo control condition and the placebo condition, *p* (unc.) < 0.001, two continuous or more voxels.

To identify the neural network of empathic pain in this experiment, we contrasted the pain pictures with the no pain pictures in the no-placebo control condition (CP-CN). Significant brain activity was detected in the postcentral gyrus, PI and MCC (see Figure 3 and Table 1).

**Table 1:** Activated regions in the contrast of (CP-CN), (CP-CN) − (PP-PN) and (PP+PN) − (CP+CN).

					MNI		
Brain regions	BA	KE	T	p(unc.)	*x*	*y*	*z*
**CP-CN**						
Right inferior parietal lobule	40	11	4.36	0.001	69	−33	27
Left Postcentral Gyrus	5	9	3.73	0.001	−36	−45	63
Left Postcentral Gyrus			3.34	0.001	−24	−45	69
Left Superior Temporal Gyrus	38	4	3.63	0.001	−45	18	−33
Left Insula	13	8	3.41	0.001	−36	−12	9
Left Middle Cingulate Gyrus	24/31	2	3.40	0.001	−15	−12	48
Left Inferior Parietal Lobule	40	4	3.32	0.001	−63	−36	24
*Left Postcentral Gyrus (cluster level, *p* = 0.092)		6	3.73	0.001	−36	−45	63
*Left Insula (cluster level, *p* = 0.049)		8	3.41	0.001	−36	−12	9
**(CP-CN) − (PP-PN)**						
Right Superior Temporal Gyrus	22	12	3.96	0.001	66	−48	18
Left Superior Temporal Gyrus	41	8	3.54	0.001	−39	−39	9
Left Superior Temporal Gyrus		2	3.52	0.001	−45	18	−33
Left Insula		3	3.35	0.001	−39	−15	9
*Left Insula (cluster level, *p* = 0.081)		2	3.35	0.001	−39	−15	9
**(PP+PN) − (CP+CN)**						
Left Inferior Frontal Gyrus_Orbitals	47	39	3.99	0.001	−36	18	−15
Left Inferior Frontal Gyrus_Orbitals			3.47	0.001	−33	27	−12
Left Inferior Frontal Gyrus_Orbitals			3.46	0.001	−39	33	−6
Left Superior Temporal Gyrus	21/38	13	3.55	0.001	−48	3	−12
*Left Inferior Frontal Gyrus_Orbitals (cluster level, *p* = 0.015)	25		3.97	0.001	−36	18	−18

Notes: for the whole-brain analyses, the threshold was set at *p* < 0.001 (voxel level, uncorrected), and two or more contiguous voxels. For the SVC analyses (noted by *), the threshold was set at *p* < 0.001 (voxel level, uncorrected, two or more contiguous voxels). CP-CN: watching pain pictures vs. watching no pain pictures, in the no-placebo control condition; (CP-PP)-(CN-PN): (no-placebo control condition vs. placebo condition, when watching pain pictures) − (no-placebo control condition vs. placebo condition, when watching no pain pictures); (PP+PN) − (CP+CN): placebo condition vs. no-placebo control condition, regardless of pain or no pain pictures.

#### Brain Regions Showing Attenuated Activity in Empathy for Pain by the Placebo Effect

To investigate whether the brain network of empathy for pain could be relieved by the placebo effect, we calculated the interaction between CONDITION and PICTURE [(CP-PP) − (CN-PN)]. Significant activation was observed in the postcentral gyrus, the PI, and the superior temporal gyrus (see Figure 3 and Table 1). In addition, we conducted a contrast test to explore whether the placebo effect also worked on no pain pictures (CN-PN) and no significant activation was found.

#### Activity in the Modulation Network of the Placebo Effect

**Figure 4:**
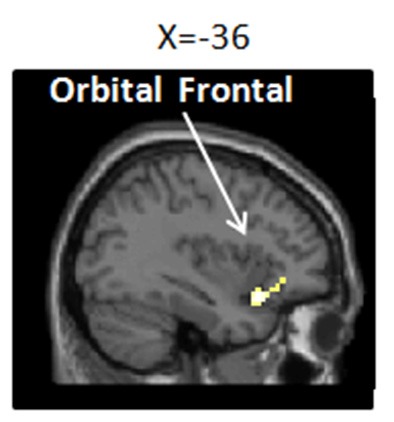
Brain network of the placebo effect of (PP+PN) − (CP+CN; i.e., placebo condition vs. no-placebo control condition, regardless of pain or no pain pictures.). Enhanced activation in the placebo treatment was found in the left inferior orbital frontal gyrus (−36, 18, −15), *p* (unc.) < 0.001, two continuous or more voxels.

To determine whether the modulation network of the placebo effect was activated, we tested the main effect of the placebo effect [(PP+PN) − (CP+CN)]. Our results showed significant activity in the OFC; see Figure 4 and Table 1.

#### Correlation Analysis

It was found that there was a significant positive correlation between changes in PI activation and changes in pain evaluation between pain and no-pain condition under the placebo modulation, brain activity was predicted by pain intensity ratings, *β* = 0.357, *p* = 0.016; no significant correlation in other contrasts was found.

## Discussion

The current study revealed the sensory regional activation which occurred in S1, S2, and the PI while watching others in pain, and for the first time, decreased activation of sensory areas (the PI) modulated by the placebo effect was observed in the neural network of empathy for pain.

Some typical regions of direct pain were found activated when observing others in pain, including the postcentral gyrus (the somatosensory cortex, S1, and S2), the PI and the MCC. In previous studies, increased activation in the somatosensory cortex and in the PI has been repeatedly reported and are widely believed to represent the sensory component of pain processing (Bushnell et al., 1999; Hofbauer et al., 2001; Bornhövd et al., 2002; Rainville, 2002). S1 and S2 are consistently considered as core regions of somatic perception and discrimination, through coding for location, strength, and quality of the stimuli. Specifically, S1 palys a role of identifying the discrimination of the stimuli and S2 is mainly responsible for the integration of the sensation messages (Treede et al., 1999). The PI connects reciprocally with the secondary somatosensory cortex and receives projections from the ventromedial nucleus (posterior part) of the thalamus that are highly specialized to convey information such as pain and temperature (Craig et al., 2000). Researchers considered that the activation in the PI represented a kind of sensory-discriminative characteristic (Brooks et al., 2002; Bingel et al., 2003). Additionally, we found significant MCC activities during empathy for pain. In terms of previous studies, the activation in this region represented the affective component of empathy (Singer et al., 2004; Jackson et al., 2006). In fact, it is believed that ACC/MCC activation was not only related with observing others in pain but also with other emotional situations, such as social exclusion (Masten et al., 2011) and disgust (Wicker et al., 2003; Jabbi et al., 2007). These findings suggest that ACC/MCC might engage in the general affective process of empathy for pain.

More importantly, for the first time, we observed decreased activation in the PI, modulated by placebo manipulation, when observing others in pain. Furthermore, this reduction merely occurred with the pain pictures instead of no pain pictures. The results are in line with previous findings in which placebo analgesia could only modulate thermal pain stimuli/negative emotion pictures representing threat/danger signals and not warm stimuli/neutral pictures (Wager et al., 2004; Zhang et al., 2011). In addition, the analysis showed that in the PI the relieved brain activation by the placebo effect was almost overlapped with the activation discovered when observing others in pain without placebo manipulation. Some evidence suggests that the activation found in the PI, induced by pain stimuli, might be highly responsive to the experience of pain. In one study, researchers applied continuously varied heat pain on subjects’ right leg and recorded their brain activation, the results showed that the only significant positive correlation between the absolute cerebral blood flow (CBF) changes and pain ratings within subjects was observed in the dorsal PI (Segerdahl et al., 2015). In another study, patients with epilepsy were given increasing thermal energy and the evoked potentials were recorded with electrodes implanted in both SII and the PI. The result showed that SII responses were more sensitive to the variation of the intensity of stimuli during the no pain level and tended to show a ceiling effect for higher pain intensities, while the PI was not able to detect innocuous stimuli but reliably tracked the dynamic changes of stimuli intensity at pain levels (Frot et al., 2006). Based on these findings, we think, this study provided more powerful evidence that the activation in sensory area, at least in the PI could be highly responsive to empathic pain in the framework of a picture-based paradigm, by using placebo manipulation to modulate empathic pain, compared to other fMRI studies that also reported sensory regional activation during empathy for pain (Jackson et al., 2006; Osborn and Derbyshire, 2010; Lamm et al., 2011).

We observed decreased subjective ratings related to empathic pain and increased activation in a restricted area to be recruited in the OFC under the placebo condition. Previous studies verified that that placebo effect could at least last for several days after the first positive experience of analgesia (Colloca and Benedetti, 2006). For those who had prior experience of the placebo effect, when the context information remains consistent, their responses are considered to be relatively reproducible (Whalley et al., 2008; Morton et al., 2009). Furthermore, repeated acquisition of conditioning enhanced the consequences of consolidation and reinforcement of the placebo belief built upon the prior experience (Benedetti et al., 1998; Colloca and Benedetti, 2006). In our study, we selected placebo responders based on their performance in a prior experiment and later examined the placebo effect in a relatively similar situation, regression analyses demonstrated that participants’ placebo performance from the prior experiment could significantly predict their later performance in the fMRI experiment to a high extent. This result demonstrates the performance of the placebo responders from the two experiments was relatively consistent. Additionally, our imaging findings in OFC were in concordance with previous research (Wager et al., 2004; Zhang et al., 2011, 2013). Evidence showed that the increased activity in OFC triggered by the placebo effect was highly related to the endogenous mu-opioid release, which greatly contributed to relieving pain perception and negative emotions (Benedetti et al., 2005; Zubieta et al., 2005; Wager et al., 2007). These findings indicated that we successfully built up the placebo effect in our research. Finally, there is some clinical implication in alleviating empathic pain by placebo modulation. Empathy may facilitate caring behaviors of medical workers but at the same time can exhaust their emotional and cognitive resources and then interfere with their ability to care for the patients (Decety, 2011; Decety et al., 2016). A learning-based placebo effect within a framework of conditional reinforcement and cognition-based reappraisal has a common anxiety-relieving effect. The learning-based placebo modulation only depends on a small recruitment of subgenual cingulate/inferior-OFC, whereas the cognition-based reappraisal usually has an enhanced mobilization of lateral prefrontal cortex resources to meet the individual’s cognitive regulation needs (Zhang et al., 2011, 2013). Therefore, if placebo manipulation carried by certain kind of equipment or drug could lead medical workers to believe it can reduce their negative arousal when seeing other in pain, then the effect of analgesic or anxiolytic effect would occur without mobilizing more cognitive resources of the dorsal lateral prefrontal cortex.

We observed significant MCC activation during empathy for pain, while failed to find decreased activities in this area under placebo modulation. On one hand, the lack of decreased activation in MCC could be largely explained by the picture-based paradigm we used. A meta-analytic study has announced that compared with the cue-based paradigm, the picture-based paradigm induced much more somatosensory regional activation other than affective related activation (Lamm et al., 2011). On the other hand, this result may be partly attributed to the decreased salience of the stimuli following the two-round experiments. Previous studies have illustrated that salience was highly related to the emotional and affective characteristics of the stimuli, which induced significant activation mainly in the ACC/MCC and AI (Downar et al., 2002; Legrain et al., 2011). As a result, compared with the sensory regional activation in the PI, activation in MCC might be much more susceptible to be affected by the significantly decreased salience of stimuli. The general decreased activation in MCC made it hard to detect whether placebo manipulation could modulate activation in MCC. Considering this evidence, here the lack of findings about altered activation in MCC being modulated by the placebo effect does not necessarily mean that MCC activity was not engaged during empathy for pain. In the future we will consider the salience of the stimuli and design an independent experiment to test whether MCC can be modulated by the transferable placebo effect.

It is worth noting the shared neural representations in the pain matrix may not be specific to the sensory qualities of pain, but instead might be associated with more general survival mechanisms such as aversion and withdrawal when exposed to danger and threat (Decety and Michalska, 2010; Decety, 2011; Decety et al., 2012). On the one hand, one study found both pain and rejection activated different multivariate patterns within their overlapped areas, indicating separable neural representations that were co-localized at the gross anatomical level (Woo et al., 2014); The critical agent of discrimination may be driven by the differences in specific activity patterns in regions activated by both physical and social pain, rather than the level of activation of a specific region (Wager et al., 2013). On the other hand, it was found that the fMRI responses triggered by nociceptive stimuli could be largely explained by multimodal neural activities (Mouraux et al., 2011); these multimodal responses are likely to reflect brain processes related to the process of detecting salient sensory events, including the most salient events of nociceptive stimuli, regardless of the sensory modality through which these events are conveyed (Legrain et al., 2011). Even so, there is a likely difference in the neural network between the threat/pain responses and orientation responses to general salient events. If the threat/pain-related responses have protective significance for survival, then they are hard to habituation; In contrast, the general salient stimuli without danger could soon get accustomed to. In the future, we may detect the neural network highly responsive to threat/pain stimuli with a repetition stimuli paradigm (Kim, 2017), through observing the activations in what brain regions by pain/empathy pain stimuli would be significantly reduced under multiple stimulus repetition and in what regions can survive.

To summarize, one contribution of this study is that we used a novel experimental design that could more definitely detect sensory regional brain activation in the process of empathy for pain. Compared with previous experimental designs, for the first time, we successfully detected alteration of the activation of sensory regions during empathy for pain by applying placebo modulation and a picture-based paradigm. This new experiment design is implicated for future research that focuses on the sensory regional activation, instead of affective ones, during empathy for pain. It is also promising to be generalized to other research topics in empathy, for example, empathy for “social pain” (social isolation). In addition, the strategy of combining the “random-controlled” instruction with enhanced placebo belief by conditioning shed some light on clinical research and interventions which want to use placebo manipulation to relieve pain as well as some mental disorders, such as Parkinson’s and depression (Andrews, 2001; de la Fuente-Fernández et al., 2001; Mayberg et al., 2002; Benedetti et al., 2004). Even though abundant evidence has already shown that the placebo effect can produce concrete treatment effects on patients apart from the actual medical treatment (de la Fuente-Fernández et al., 2001; Fountoulakis and Möller, 2011; Benedetti, 2014), it is still quite controversial to apply the placebo modulation as a kind of treatment into a clinical situation. One dilemma is that the deceptive instructions had strong placebo power but does not fit the clinical ethical standards. This study revealed a significant placebo effect by avoiding directly deceptive instructions (e.g., the effective treatment might be applied to you or not) and combining corresponding reinforcement conditions (e.g., if you felt pain relieved then you were supposed to belong to the treatment group).

One limitation of the study is that we did not measure other factors affecting placebo effect, such as expectation level and personality traits, which are both very important (Frisaldi et al., 2018). For example, expectations would predict placebo effect independently of personality factors and highly correlate to placebo effects (Corsi and Colloca, 2017; Frisaldi et al., 2017, 2018). Participants’ performance on placebo manipulation was found to be correlated with some personality traits, such as suggestibility, acquiescence, dispositional optimism, and resiliency (Corsi and Colloca, 2017; Frisaldi et al., 2018). We will add these measurements in future research in order to pursue multi-faceted evidence on the reliability of the placebo effect and placebo responders. In addition, the brain signals were not recorded when participants were perceiving pain in self and in others in the behavioral experiment, due to the incompatibility of the pain stimulator and the MRI scanner. If activation of the sensory area is relieved in both sessions, then such evidence will be more persuasive regarding whether this activation is pain essential and is shared by empathy for pain. Furthermore, we found significant activation in the MCC during empathy for pain while failing to detect reduced activation in the same region by placebo manipulation. One reason may be that repeated training in the behavioral and fMRI experiments reduced participants’ sensitivity to pain pictures. In future studies we will scan brain activities not only in the empathy for pain phase but also during pain in self-phase in the both behavioral and fMRI experiments, thus we may observe whether activation of the sensory area will be relieved in both sessions and whether neural activities such as MCC will be decreased due to repeated trainings. Finally, the present study included more females participated in the task which is twice as many as males both in behavioral experiments and in the fMRI experiment, so it might bring the effect of gender bias. In the future study, we would employ an equal number of females and males to exclude gender effect.

## Conclusions

In our study, we observed a significant activation in both the sensory areas (the postcentral gyrus and the PI) and MCC during empathy for pain. More importantly, for the first time, we found that placebo modulation could relieve sensory activity in the PI during empathy for pain, which allows for a powerful inference that the reduced activities of the PI sensory areas induced by the placebo effect can be attributed to the empathy for pain itself. By using placebo modulation, this study provides a relatively new approach for future research that focuses on the sensory components during empathy for pain.

## Data Availability Statement

The datasets analyzed in this article are not publicly available. Requests to access the datasets should be directed to WZ, zhangwc@psych.ac.cn.

## Ethics Statement

The studies involving human participants were reviewed and approved by the ethics committee of the Chinese Academy of Science. The patients/participants provided their written informed consent to participate in this study.

## Author Contributions

WZ, JL, and JZ contributed to the conception and study design. YZ and RL organized the database and wrote sections of the manuscript. YZ performed the statistical analysis. YZ and WZ wrote the first draft of the manuscript. All authors contributed to manuscript revision, read, and approved the submitted version.

## Conflict of Interest

The authors declare that the research was conducted in the absence of any commercial or financial relationships that could be construed as a potential conflict of interest.

## Footnotes

^1^https://www.rstudio.com/

^2^http://www.fil.ion.ucl.ac.uk/spm

^3^https://www.nitrc.org/projects/wfu_pickatlas/
